# AbThera, Botox, and Fasciotens: A Trifecta in Open Abdomen Management

**DOI:** 10.7759/cureus.72829

**Published:** 2024-11-01

**Authors:** Phi Nguyen, Ramana Balasubramaniam

**Affiliations:** 1 General Surgery, Goulburn Valley Health, Shepparton, AUS; 2 Institute of Minimal Access, Metabolic and Bariatric Surgery (iMAS), Sir Gangaram Hospital, New Delhi, IND; 3 Abdominal Wall Reconstruction, Clinic Ramana, Kolkata, IND

**Keywords:** abthera, botox, botulinum toxin a, fascia retraction, fasciotens, negative pressure wound therapy (npwt), open abdomen, vertical traction device

## Abstract

The management of patients with open abdomen (OA) has long been a frustrating problem for surgeons, with high morbidity and mortality. OA secondary to laparotomy for septic peritonitis (one of the commonest causes) requires the control of abdominal wall retraction, prevention of evisceration and bowel fistulae, and overall control of infection. We present here the successful implementation of a relatively novel therapeutic combination of three different modern interventions on a 68-year-old patient with an open abdomen caused by an anastomotic leak following the reversal of Hartmann’s operation. We were successful in primarily closing the abdominal wall after 14 days.

## Introduction

Patients with an open abdomen (OA) are at high risk of major complications, including multiple organ failure, intraabdominal abscesses, enterocutaneous fistulae, loss of abdominal domain, and development of complex abdominal wall hernias [1]. Expert consensus is that the early closure of OA should be a major goal in the treatment of OA. When abdominal closure is not possible in the distended peritonitic state, temporary closure needs to be considered to prevent the complications of abdominal wall dehiscence (burst abdomen) and abdominal compartment syndrome (ACS) [2]. An ideal method of temporarily closing the abdomen should protect the abdominal contents, prevent further contamination, preserve abdominal wall tissue, avoid fascial retraction, prevent ACS, and facilitate early abdominal closure. Recent advances in wound care management, as well as the improving understanding of abdominal wall biomechanics, have led to the use in OA management of mesh-mediated fascial traction (MMFT), Botulinum toxin A (Botox, Allergan Aesthetics, Irvine, California, US), and wound protection systems like AbThera (KCI, San Antonio, Texas, US). Each of these plays a different and synergistic role in OA management. Botox paralyzes the lateral abdominal wall muscles and reduces the fascial retraction of the OA wound. AbThera (or its comparable peers) protects the intraabdominal viscera from the overlying dressings and ensures suction evacuation of exudate, pus, or other fluids, thereby reducing the intraabdominal septic volume and the intraabdominal pressure (IAP). Mesh-mediated fascial traction in a horizontal direction has been used in recent times to bring the fascial edges together over a period of time to allow subsequent closure in the same admission. However, this approach is now been superseded by a vertical traction method (fasciotens; Fasciotens GmbH, Essen, Germany).

Our case here demonstrates the use of AbThera, Botulinum Toxin A, and Fasciotens in the management of a patient with an open abdomen in the management of generalized peritonitis. The sepsis state was induced by an anastomotic leak after the reversal of a Hartmann operation previously performed for perforated sigmoid diverticulitis. Despite several setbacks, abdominal closure was completed safely, quickly, and effectively.

## Case presentation

**Figure 1:**
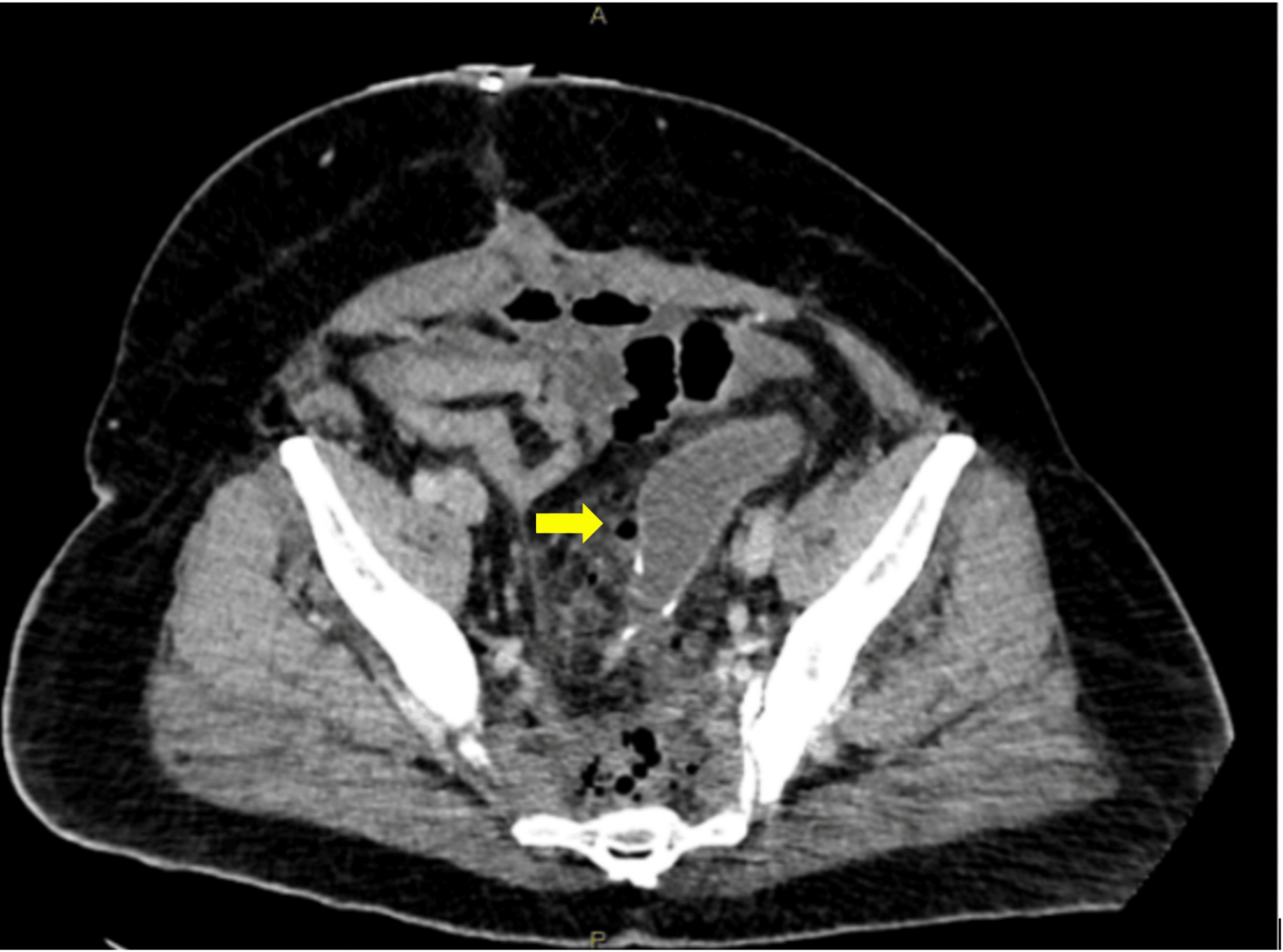
Axial CT abdomen pelvis showing gas locules adjacent to the anastomosis (yellow arrow)

**Figure 2:**
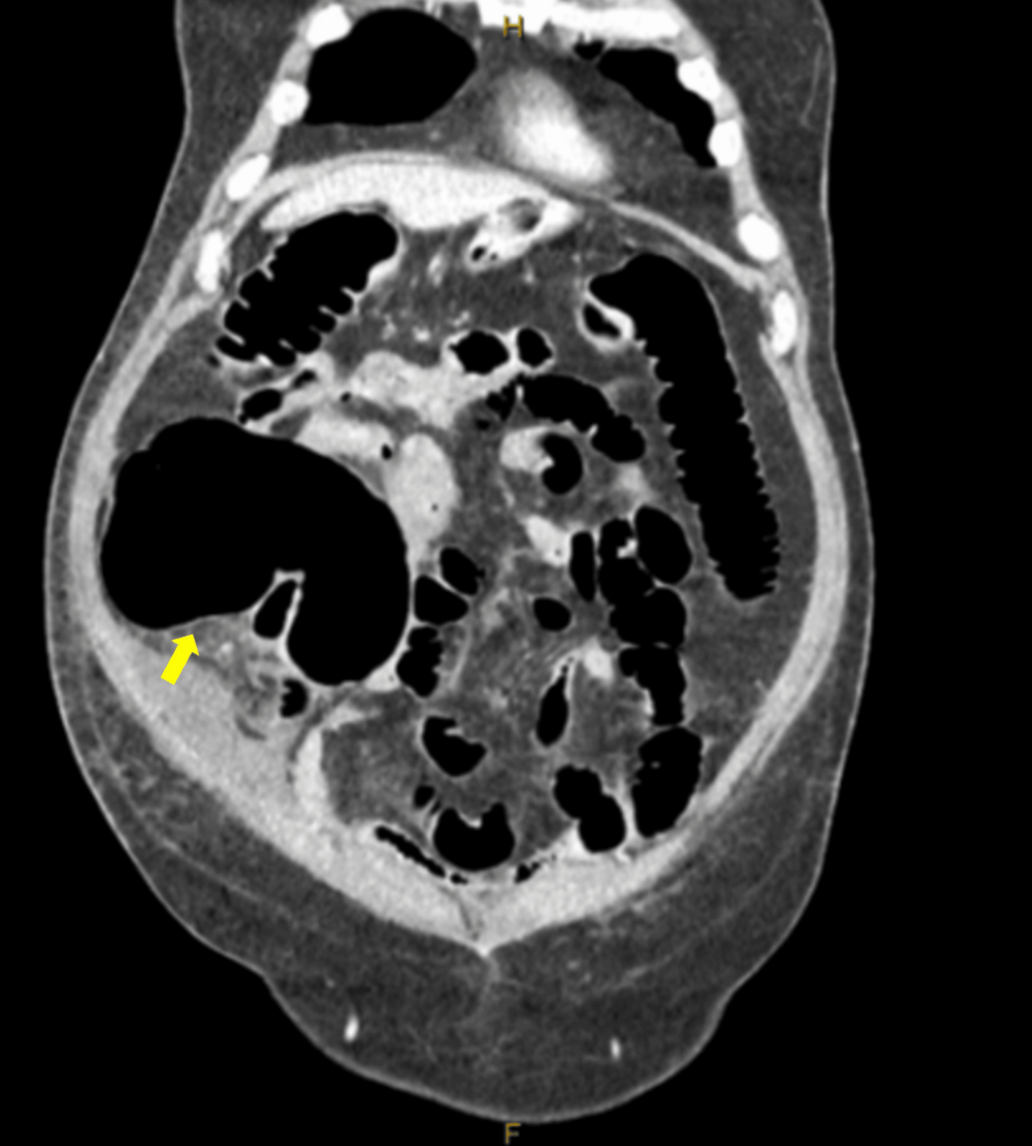
Coronal CT abdomen pelvis demonstrating a distended right colon, especially the cecum (yellow arrow)

A 68-year-old male underwent an elective reversal of Hartmann in a regional hospital in Victoria, Australia, following previous perforated diverticulitis one year ago. The reversal of Hartmann was a technically difficult operation. There was ample length of the rectal stump, and it was anastomosed to the descending colon. Upon completion of the operation, flexible sigmoidoscopy demonstrated a healthy anastomosis and a negative leak test. On postoperative day 5, the patient experienced increasing abdominal pain and fever. A CT scan of the abdomen and pelvis revealed gas locules in the mesorectum and presacral space (Figure 1). The colon was grossly distended akin to a closed-loop obstruction (Figure 2). These findings suggested intraabdominal sepsis secondary to an anastomotic leak.

Broad-spectrum antibiotics were commenced. An exploratory laparotomy revealed four quadrant fecal peritonitis. The colorectal anastomosis was found to have dehisced. Additionally, the ascending colon was ischemic and perforated at four different locations. The colorectal anastomosis was dismantled, the proximal end brought out as an end colostomy and the rectal stump closed with absorbable sutures. Right hemicolectomy was performed to resect the diseased colon until the hepatic flexure and the terminal ileum exteriorized as a spouted end Brooke stoma. Extensive lavage was performed. Histopathology subsequently revealed the proximal colon to have sustained full thickness perforations with ischemic features.

**Figure 3:**
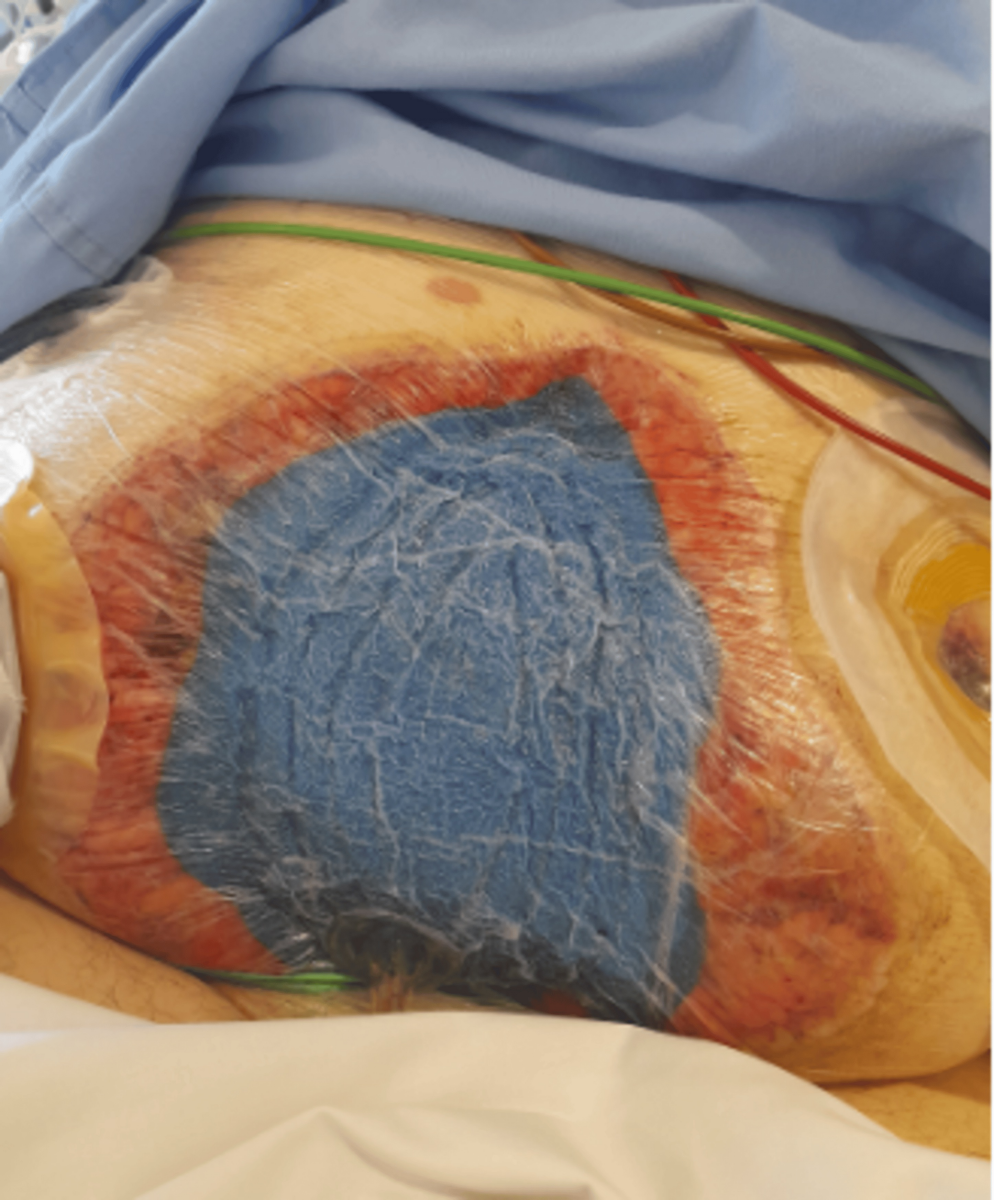
Clinical photograph of the open abdomen with AbThera after the first relook laparotomy

Due to pronounced edema of the bowel, the abdomen was left open. There were no options for fascial closure at this point in time. In addition, relooks in the theater for lavage were needed to clean up the contents from the peritoneal cavity. The initial fascial distance measured 18 cm under full relaxation. The AbThera system was applied (Figure 3) and the patient was admitted to the intensive care unit.

A planned relook was performed the following day. One day prior to the first relook, using the ultrasound, the surgeon identified the external oblique, internal oblique, and transversus abdominis muscles and injected 50 units of Botox at three separate points on each side of the abdominal wall. The patient received a total of 300 units of Botox to the abdominal wall.

**Figure 4:**
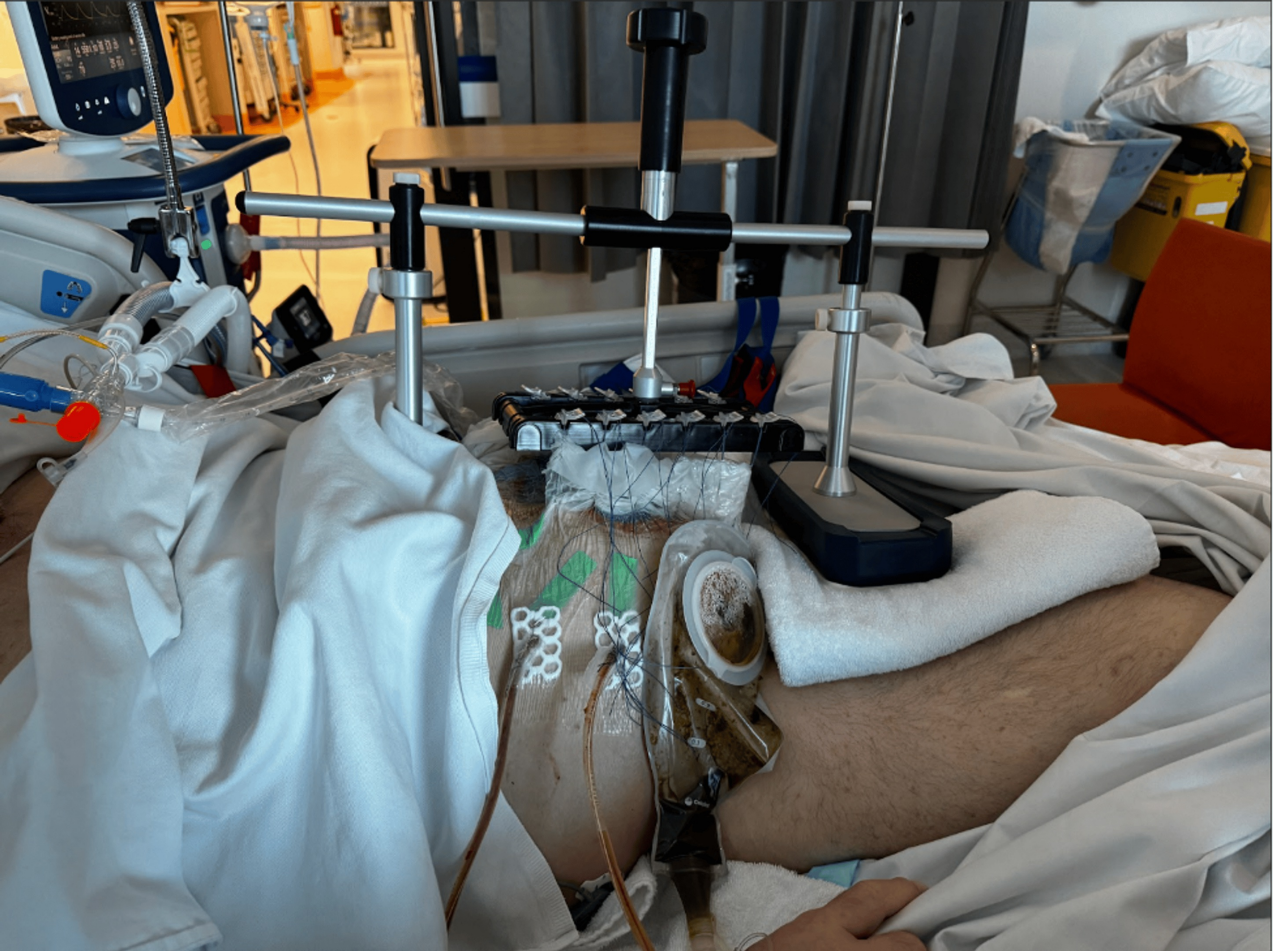
Clinical photograph of the abdomen after the first Fasciotens application and ileostomy

Two days from the first relook, the second relook revealed no further ischemia or peritonitis. The AbThera sheet and foam were placed as usual to completely cover the peritoneal cavity. Two polypropylene meshes were then affixed to the anterior sheath with a running PDS suture. Six No. 2 Vicryl sutures were applied to each side of the mesh at equal intervals and connected to the Fasciotens abdomen device for traction. The sutures were securely sealed within two layers of adherent plastic sheets on either side and crossed over to the other side. The crossing of the traction sutures enables the device to pull diagonally as well as vertically on the abdominal wall, increasing the fascial approximation. The device was assembled and placed on the chest and anterior pelvis (Figure 4). The product was aligned according to the manufacturer’s recommendation based on defect location and size. Postoperatively, during the daytime, a load of 80 Newtons was applied to the abdominal wall. Treatment periods were five hours long with intermittent rest periods of one hour each. The application of Fasciotens required education for staff. The device was applied during the period when the patient was intubated in an intensive care unit setting.

During the remaining course of his inpatient stay, four additional relooks were carried out over the course of 14 days. There were concerns arising from ileostomy die-back needing revision and the increasingly frozen abdomen. In addition, he developed cellulitis and collection in the right flank owing to the necrosis of the stoma. The fascial distance reduced significantly, from 18cm to 5cm, over this time. On the 14th day of the Fasciotens treatment, the abdominal wall closure was completed using a 1.0 PDS figure of eight sutures in an interrupted fashion.

**Figure 5:**
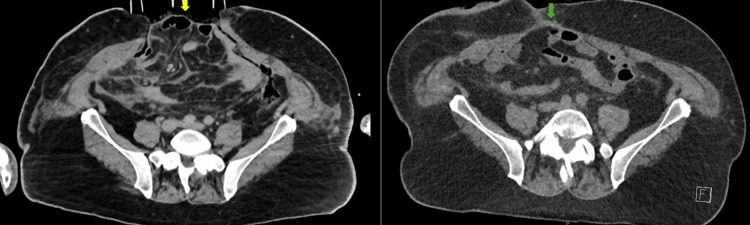
Axial CT abdomen pelvis (left) indicated a fascia defect (yellow arrow) on day 1 post application of the Fasciotens. CT abdomen pelvis (right) indicated successful primary fascia closure (green arrow) prior to discharge.

In addition to the above, the rectal stump also leaked, leading to a persisting presacral collection and low-grade sepsis. It was managed with the sigmoidoscopic insertion of an endosponge. This was removed five days afterward. The patient had a total of 12 weeks of acute inpatient stay and was discharged to a rehabilitation center. Before discharge, a CT scan was performed showing an intact abdominal wall (Figure 5). To the point of submitting this report, he has not developed any signs of an incisional hernia. 

## Discussion

A key challenge for the treatment of patients with open abdomen is to prevent fascia retraction. Currently, there are several techniques for temporary abdominal closure systems including simple packing, vacuum packing, Wittmann-Patch, silo techniques, and negative pressure wound therapy (NPWT). Their primary fascial closure rates are heterogenous with reported rates of 69 to 92% [3-6].

Historically, the vacuum pack technique was introduced by Brock in 1995. It involves a three-layer system: a polyethylene sheet between the viscera and abdominal wall, a moist surgical towel with two suction drains, and an adhesive drape covering the wound, with continuous negative pressure of 100-150 mmHg applied. It has 52% primary fascial closure rates [7]. Vacuum-assisted fascial closure techniques emerged later, using polyurethane sponges and vacuum pumps, achieving higher fascial closure rates up to 88%. The Velcro adhesive sheet technique, developed in 1990 and later commercialized as Wittmann-Patch®, uses two biocompatible polymer sheets with hooks and loops for gradual abdominal wall closure. The sheets are sutured to opposite fascial edges, compressed together, covered with a surgical towel, and connected to suction. It has a 90% primary fascia closure rate [7]. Meanwhile, the Botoga bag technique, which involves suturing a 3-L irrigation bag to the fascia, did not show preservation of the fascia or prevent intra-abdominal hypertension [8].

This case presented the effective treatment of an open abdomen with a combination of the utilization of AbThera, Botox, and Fasciotens. Between the first and last relook laparotomies, we observed a significant reduction of the gap between both fascial margins from 18 cm to 5 cm over 14 days.

AbThera is a negative pressure wound therapy device. It is made of an oval-shaped polyurethane film containing an absorbent sponge with six extended arms. It has a large visceral surface area coverage that allows coverage of the entire peritoneal cavity from the diaphragm to the pelvis and one paracolic gutter to the other. It is designed to remove fluid, reduce edema, provide medial tension (which minimizes fascial retraction and loss of domain), and facilitate the isolation of the viscera and abdominal contents from the external environment. It also ensures separation between the abdominal wall and the viscera, thereby protecting the abdominal contents. The six arm extensions of polyurethane film enhance its ability to reach the different recesses of the cavity and allow for enhanced peritoneal fluid drainage, which reduces the septic burden.

Botulinum toxin A (BTA) was utilized as an adjunct in this case in the management of the open abdomen. BTA acts by binding to pre-synaptic cholinergic nerve terminals and decreasing the release of acetylcholine, causing a neuromuscular blocking effect. BTA takes at least two to three days to demonstrate any effect and up to three weeks to reach maximum effect. In this case study, BTA allowed relaxation and elongation of the abdominal wall muscles, thereby improving the chances of primary fascial closure. Previous reports show a rate of primary fascia closure ranging from 52% to 83% [9-11]. A multi-institutional, prospective study of Level I trauma centers in 2013 indicated a primary fascial closure rate of 66% [12]. However, a systemic review by Luton et al. of 14 studies investigating the rate of delayed primary fascia closure of the open abdomen in the emergency setting following injection of BTA into the lateral abdominal wall. The quality of evidence is poor due to a small number of trials and based on high levels of bias found between trials. Therefore, it does not provide strong enough evidence to either advocate routine BTA injection in this setting or to advise against it [13].

**Figure 6:**
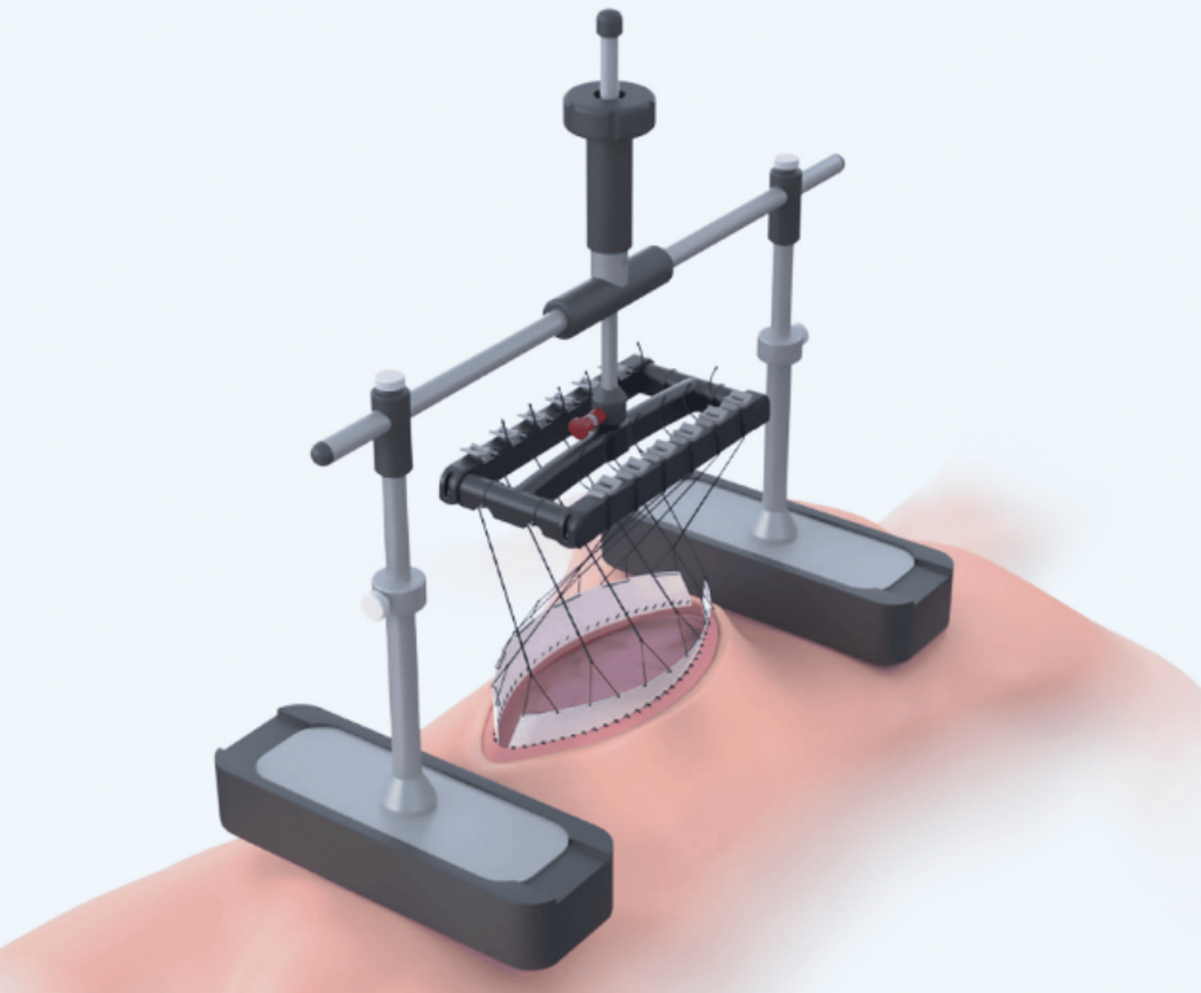
OptiMed Fasciotens OptiMed Technologies, North Rocks, Australia

In this case study, Fasciotens (Figure 6) was found to be useful in preventing fascial traction by applying vertical traction while the abdomen was left open. Compared to the horizontal traction method, vertical traction is considered superior in the management of open abdomen cases due to its immediate applicability. Unlike horizontal traction, which is often hindered by the presence of intestinal edema during the initial phase of open abdomen care, vertical traction of Fasciotens can be applied immediately. A key function of vertical traction of Fasciotens is its ability to prevent the retraction of the lateral abdominal muscles. By applying vertical tension, the muscles are held in place, maintaining their natural positioning, which facilitates a faster fascial closure process. Without vertical traction, muscle retraction may progress unchecked. Another significant advantage of vertical traction is its ability to increase the volume of the abdominal cavity, thereby reducing intra-abdominal pressure. High intra-abdominal pressure, if uncontrolled, can lead to abdominal compartment syndrome, which negatively impacts multiple organ systems, particularly the respiratory, renal, and circulatory systems.

Currently, there is limited evidence of Fasciotens for the management of the open abdomen in the literature. A study by Fung et al. demonstrated Fasciotens significantly reduced fascia-to-fascia distance after 48 hours of application and primary fascia closure was achieved at a mean period of 7 days [14]. The primary limitations of this study include its retrospective design and the small sample size of only 20 patients. There was no control group to compare the outcomes of Fasciotens with other techniques, making it difficult to definitively attribute the results to the use of the Fasciotens alone. The study also lacks long-term follow-up data on patient outcomes, particularly regarding the development of complications such as incisional hernias. Another case report by Mavc & Kunz demonstrated the additional benefits of Fasciotens, including rapid improvement of respiratory dynamics, diuresis, stoma output, and hemodynamics of the patient [15]. Similarly, the absence of a control group and the lack of long-term follow-up emphasizes the need for further research on Fasciotens. This calls for the need for multicenter randomized trials, long-term outcome studies, and cost-effectiveness analysis of Fasciotens.

## Conclusions

In conclusion, the management of an open abdomen is challenging. The implementation of a trifecta of AbThera, Botox, and Fasciotens allowed early and successful closure of the abdomen while preventing prolonged ICU stay and morbidity. This combination enabled effective fascial closure and has avoided early incisional hernia along the median laparotomy. 
